# Supplementary material for: TRPV4 activation by TGFβ2 enhances cellular contractility and drives ocular hypertension
Source: eLife. 2025 Jun 24;14:RP104894. doi: 10.7554/eLife.104894 (PMC12187138; doi:10.7554/eLife.104894)
Supplement: Figure 1—source data 1. [file elife-104894-fig1-data1.pdf]

TRPV4 (Membrane)

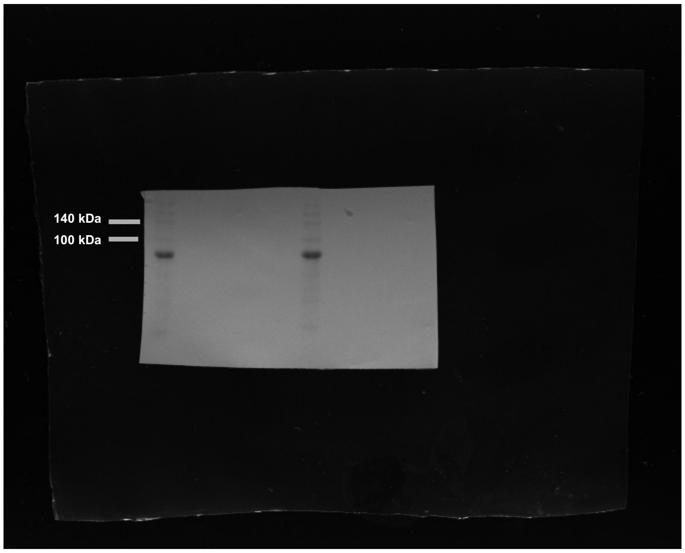

TRPV4 (Chemiluminescence)

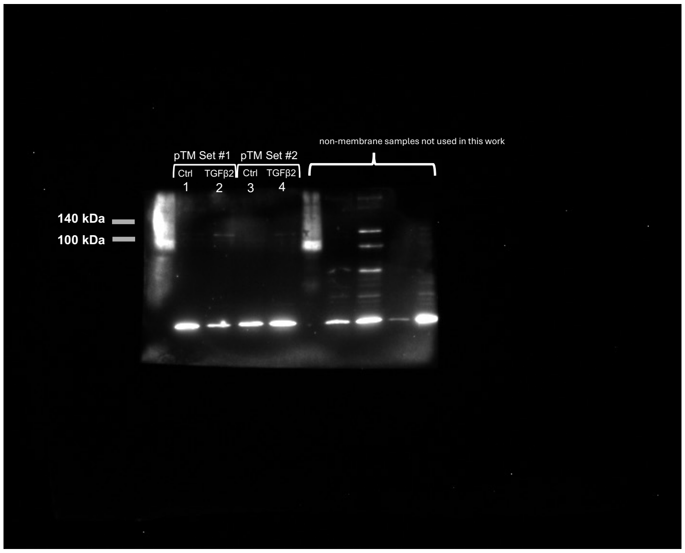

Beta-Tubulin (Membrane)

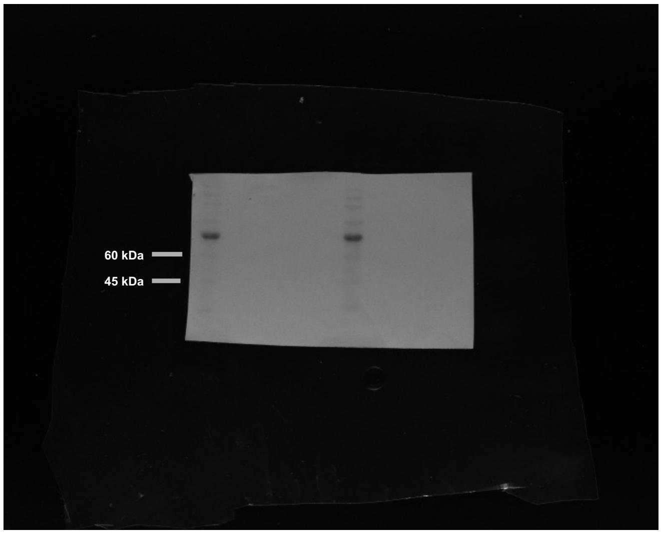

Beta-Tubulin (Chemiluminescence)

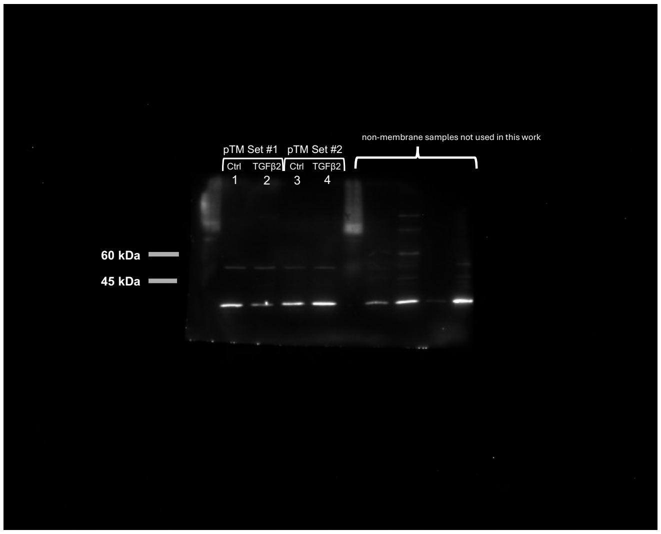

Figure 1, Source Data 1. Labeled uncropped images of the membrane and HRP-signal for the western blots shown in Figure 1 and Figure 1, Source Data 2. A molecular weight ladder and the Grouped pTM membrane samples take up the left 5 lanes of the gel. These samples were run simultaneously with varied non-membrane samples that are not included in this work, which encompass the right five lanes. The approximate location of the molecular weight markers above and below the bands of interest are labeled on each figure.
