# Supplementary figures and images for: TRPV4 activation by TGFβ2 enhances cellular contractility and drives ocular hypertension
Source: eLife. 2025 Jun 24;14:RP104894. doi: 10.7554/eLife.104894 (PMC12187138; doi:10.7554/eLife.104894)

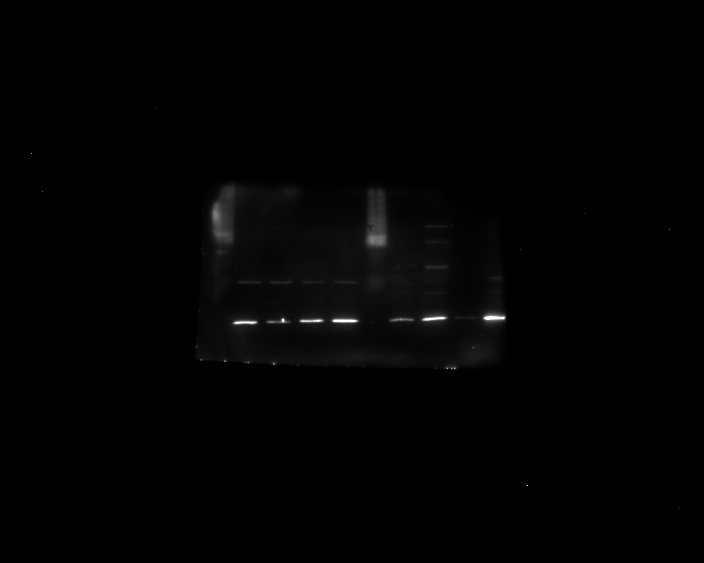

Supplement: Figure 1—source data 2. [file elife-104894-fig1-data2.zip › Figure 1-Source Data 2/Raw B-Tubulin Blot.tif]

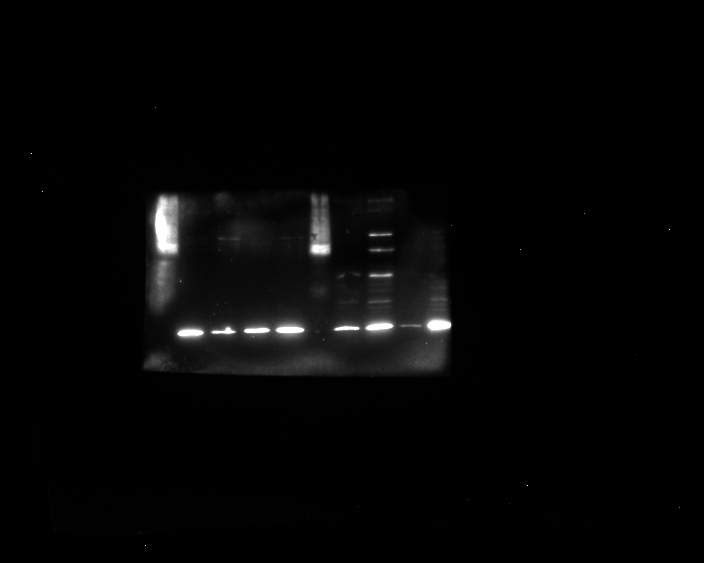

Supplement: Figure 1—source data 2. [file elife-104894-fig1-data2.zip › Figure 1-Source Data 2/RAW TRPV4 Blot.tif]
